# Cupric Oxide Nanostructures from Plasma Surface Modification of Copper

**DOI:** 10.3390/biomimetics4020042

**Published:** 2019-06-25

**Authors:** Hernando S. Salapare, Juvy A. Balbarona, Léo Clerc, Pierre Bassoleil, Arnaud Zenerino, Sonia Amigoni, Frédéric Guittard

**Affiliations:** 1 Université Côte d’Azur, NICE Lab, IMREDD, 06100 Nice, France; lclerc@outlook.fr (L.C.); pa.bassoleil06@gmail.com (P.B.); Arnaud.ZENERINO@univ-cotedazur.fr (A.Z.); Sonia.AMIGONI@univ-cotedazur.fr (S.A.); Frederic.GUITTARD@univ-cotedazur.fr (F.G.); 2 Faculty of Education, University of the Philippines Open University, Los Baños 4030, Laguna, Philippines; 3 Department of Mechanical Engineering, College of Engineering University of the Philippines Diliman, Quezon City 1101, Philippines; jabalbarona@gmail.com; 4 Department of Bioengineering, University of California Riverside, Materials Science and Engineering Building, 900 University Avenue, Riverside, CA 92521, USA

**Keywords:** cupric oxide, copper, bio-inspired material, nanostructures, oxygen plasma, surface modification, hydrophilic

## Abstract

Taking inspiration from the hydrophilic and superhydrophilic properties observed from the nanostructures present on the leaves of plants such as *Alocasia odora*, *Calathea zebrina*, and *Ruelia devosiana*, we were able to synthesize cupric oxide (CuO) nanostructures from the plasma surface modification of copper (Cu) that exhibits hydrophilic and superhydrophilic properties. The Cu sheets were exposed to oxygen plasma produced from the P300 plasma device (Alliance Concept, Cran-Gevrier, France) at varying power, irradiation times, gas flow rates, and pulsing duty cycles. The untreated and plasma-treated Cu sheets were characterized by contact angle measurements, scanning electron microscopy (SEM), and energy dispersive spectroscopy (EDS) to determine the changes in the surface of Cu before and after plasma treatment. Results showed that plasma-treated Cu sheets exhibited enhanced wetting properties compared to untreated Cu. We attributed the decrease in the measured water contact angles after plasma treatment to increased surface roughness, formation of CuO nanostructures, and transformation of Cu to either CuO_2_ or Cu_2_O_3_. The presence of the CuO nanostructures on the surface of Cu is very useful in terms of its possible applications, such as: (1) in antimicrobial and anti-fouling tubing; (2) in the improvement of heat dissipation devices, such as microfluidic cooling systems and heat pipes; and (3) as an additional protection to Cu from further corrosion. This study also shows the possible mechanisms on how CuO, CuO_2_, and Cu_2_O_3_ were formed from Cu based on the varying the plasma parameters.

## 1. Introduction

In past decades, the mimicking of nature has been very successful in solving global environmental, material, and sustainability problems due to the superior physical and chemical properties exhibited by different animals, plants, and microorganisms [1,2,3,4,5,6,7,8]. As an example, leaves of plants such as *Alocasia odora*, *Calathea zebrina*, and *Ruelia devosiana* show nanostructures that exhibit hydrophilic and superhydrophilic properties [6,7]. Aside from the surface morphology, the surface and bulk chemistry of the leaves of these plants also contribute to the exhibited wetting properties [6]. Mimicking these nanostructures to attain the same wetting properties can be achieved using different techniques, such as surface modification of polymeric or metallic materials by flame, chemical, laser, or plasma processes [1,2,3,4,5,6,7,8,9,10]. 

In recent years, plasma technologies have been widely used, due to its ability to modify the surface chemistry and surface morphology of a material by taking advantage of the presence of complex mixtures of ions, electrons, atoms, and radical species in the plasma [11,12]. 

Copper (Cu) metal and its oxides, such as cupric oxide (CuO) and cuprous oxide (Cu_2_O) has been widely studied, due to its chemical and physical properties that makes it advantageous in such applications as sensing, energy, electronics, tubing, and aeronautical industries [13,14,15,16,17,18,19,20,21]. Hydrophilic CuO nanostructures on Cu are useful in terms of its possible applications, such as: (1) in antimicrobial and anti-fouling tubing; (2) in the improvement of heat dissipation devices, such as microfluidic cooling systems and heat pipes; and (3) as an additional protection to the Cu from further corrosion [13,14,15,22]. Much research has been done on the formation of CuO and Cu_2_O by thermal oxidation [23,24,25,26,27], but only a few presents the use of plasma technology. Some of these studies are the functionalization of Cu surfaces by plasma treatments in improving the adhesion of epoxy resins [16] and the effects of different gases in the plasma treatment of the Cu surface of capacitors [17]. Although the applications of CuO formations from the plasma treatment of Cu have already been reported in the literature [16,17,28,29], there is still a need to explain the mechanisms on how varying the plasma parameters affects the formation of CuO and Cu_2_O.

In this study, we used oxygen plasma with varying plasma parameters (power, irradiation time, gas flow rate, and pulsed plasma duty cycle) to form CuO and Cu_2_O directly from Cu. The wetting properties of the plasma-treated Cu samples were explained by the changes in the surface roughness, surface morphology, and the formations of CuO and Cu_2_O. The mechanism on the formations of CuO, Cu_2_O, and the intermediate-phase Cu_2_O_3_ was explained based on the varying plasma parameters. 

## 2. Materials and Methods

### 2.1. Materials and Plasma Surface Modification

Cu sheets (CAS 7440-50-8: 99.35% purity, 63.55 MW, 0.034 mm thickness) were obtained from GoodFellow, France. The samples were cut into 1 cm × 3 cm sizes and used without further cleaning.

**Figure 1:**
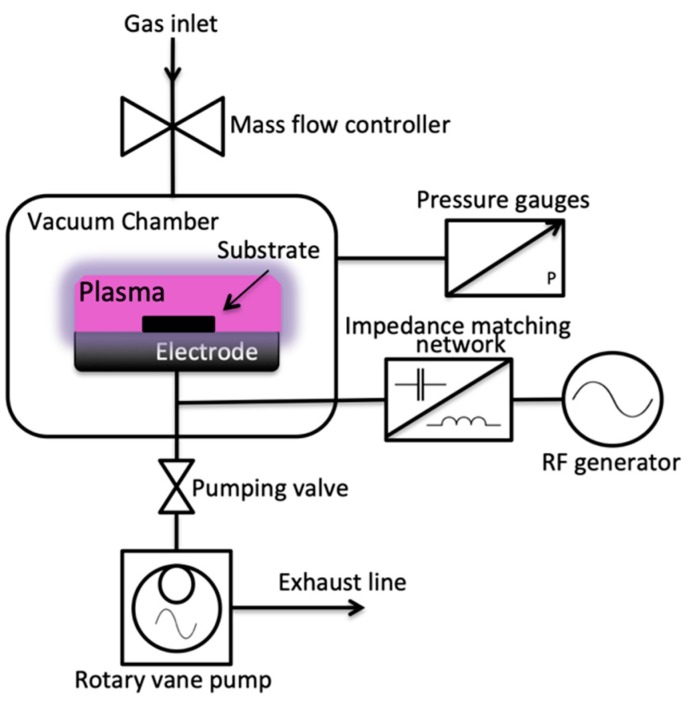
Schematic diagram of the P300 plasma device.

The Cu samples were irradiated with oxygen plasma produced from a P300 plasma device (Alliance Concept, Cran-Gevrier, France). Figure 1 shows the schematic diagram of the P300 plasma device with a vacuum chamber of 300 mm × 300 mm × 300 mm dimension, an electrode with a diameter of 10 cm, and operation at an excitation frequency of 13.56 MHz. The chamber was first evacuated using a 20 m^3^/h rotary pump, and the base pressure was achieved at 8.0 × 10^−2^ mbar before plasma ignition. Oxygen gas was then injected inside the vacuum chamber at varying flow rates of 10 sccm, 15 sccm, and 20 sccm at operating pressures of 1.55 × 10^−1^ mbar, 1.97 × 10^−1^ mbar, and 2.24 × 10^−1^ mbar, respectively. The pulsing duty cycles were varied from 0%, 50%, and 80%. Power and irradiation times were also varied from 100 W, 300 W, and 600 W, and 60 s, 180 s, and 300 s, respectively. Table 1 shows the summary of the plasma surface modification parameters.

### 2.2. Characterizations

#### 2.2.1. Water Contact Angle Measurements

Changes in the wettability of the untreated and plasma-treated Cu samples were determined using a DSA-30 goniometer (Krüss GmbH, Hamburg, Germany) equipped with drop shape analysis (DSA4) software (Krüss GmbH). The sessile drop method was utilized to determine the apparent contact angles. Two μL of Milli-Q deionized water was dropped vertically onto the samples using a motorized syringe mechanism. For each sample, water contact angles were measured at five different sites, and the standard deviation of the contact angle measurements at different sites were all statistically the same. All contact angle measurements were recorded at the same humidity and temperature conditions. Water contact angle measurements were performed 0 days, 30 days, and 60 days after plasma treatment to determine the aging effects on the Cu samples.

#### 2.2.2. Scanning Electron Microscopy (SEM)

The surface morphology of the untreated and plasma-treated Cu samples were obtained using a Phenom ProX SEM (Phenom World B.V., France Scientifique, Saint Genis Laval, France) at 15 kV accelerating voltage and at a secondary electron detector (SED) imaging mode. The SEM micrographs were reported at 10,000× magnification.

#### 2.2.3. Energy Dispersive Spectroscopy (EDS)

The elemental compositions of the untreated and plasma-treated Cu samples were determined from the 15 kV analysis mode of the SEM described in the preceding section. The detector used in the EDS is a Silicon Drift Detector (SDD) with an ultra-thin silicon nitride (Si_3_3N_4_4) X-ray window that allows the accurate detection of elements from B to Am, with an energy resolution of Mn Kα ≤ 137 eV, and with a resolution limit of ≤14 nm. Nanostructures on the plasma-treated Cu samples were examined at 10 kV analysis modes in order to have more accurate counts at dimensions of less than 1 μm. Phenom ProSuite v2.8.2 Element Identification Software (Phenom World B.V., France Scientifique, Saint Genis Laval, France) was used to calculate the atomic concentration of the elemental components using the SED mode. All the EDS calculations are within ±15% error from the theoretical values. 

## 3. Results and Discussion

### 3.1. Water Contact Angle Measurements

The water contact angle measurements are shown in Table 1 with the corresponding plasma surface modification parameters. It can be observed that all plasma-treated Cu samples exhibited enhanced wetting properties. The sample became hydrophilic at lower power and exhibited superhydrophilic properties at higher power. The decrease in water contact angle as the power increases is attributed to the increase in interactions of the different plasma species with the samples, which either leads to an increase in the surface roughness, or changes in the chemistry of the material [7,10]. 

When the irradiation times were varied while maintaining the power at 300 W, all the plasma-treated Cu samples exhibited superhydrophilic properties. This means that as long as the power is high, lesser time is needed to achieve superhydrophilic properties. 

Increasing the gas flow rate also increases the wetting properties of the samples. A lower gas flow rate produced hydrophilic surfaces, while a higher gas flow rate exhibited superhydrophilic properties. Increasing the gas flow rate increases the pressure inside the chamber, meaning that there are more plasma species that are produced that can interact with the samples. 

Changing the pulsing duty cycle of the plasma strongly modifies the plasma that interacts with the sample. A lower duty cycle means less dissociated plasmas, which means that the chemical effects of the plasma are greater than the physical consequences which occur to the sample. This was exhibited by obtaining hydrophilic surfaces at a lower duty cycle, while superhydrophilic properties were obtained at a higher duty cycle. The physical effects of the plasma on the surface, like etching, increasing surface roughness, or producing nanostructures are more enhanced when the duty cycle is higher. A lower duty cycle of pulsed plasmas reduces the physical damage that the plasma can bring to the sample. Normally, a lower duty cycle is favorable only when the etching of a material is desired [30,31,32].

Water contact angles measurements were also performed 30 days and 60 days after plasma treatment, as shown in Table 1, where the rate of hydrophobic recovery is visibly very slow and the changes of contact angles over time are insignificant. This means that the copper oxides formed were stable and favorable for future industrial applications.

### 3.2. Scanning Electron Microscopy (SEM)

**Figure 2:**
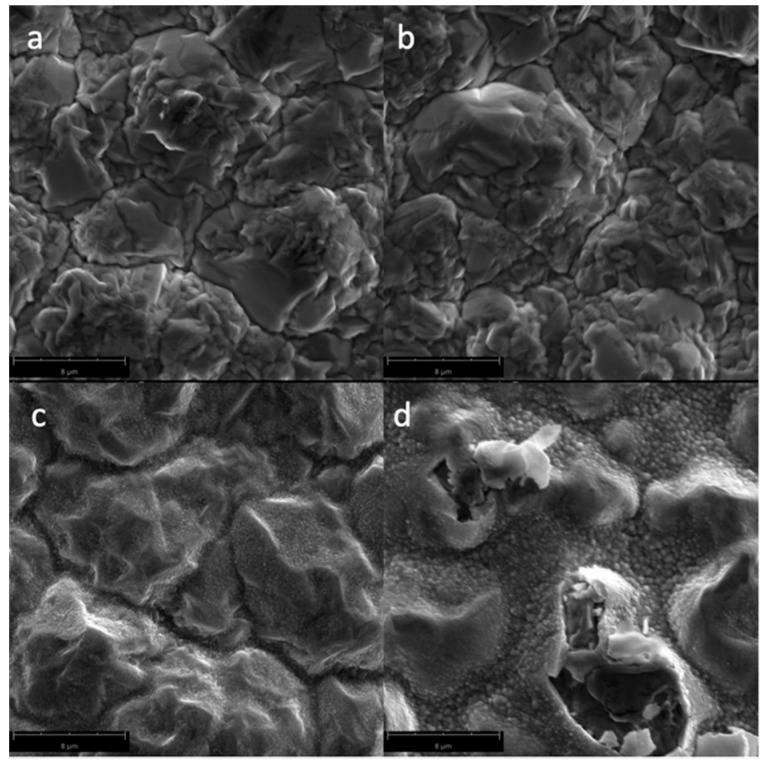
Scanning Electron Microscopy (SEM) micrographs of the surfaces of the Cu samples (magnified at 10,000×) subjected to oxygen plasma with varying powers: (**a**) untreated; (**b**) 100 W; (**c**) 300 W; and (**d**) 600 W.

Figure 2 shows the SEM micrographs of the Cu samples subjected to oxygen plasma with varying power. A smoother surface morphology can be seen for the untreated Cu samples, while plasma-treated Cu samples exhibited increased surface roughness, especially for higher power plasma treatments where CuO nanostructures (with an average diameter of 106 nm) were formed on the surface. In Figure 2d, it can be seen that some of the surface layer is bursting at 600 W plasma treatment, which may be due to oxygen gases trapped in the surface because of the thermal oxidation of Cu. The increase in the surface roughness and the presence of the nanostructures in the surface of the material is responsible for the superhydrophilic properties at higher power plasma treatments. Plasma treatment at 600 W resulted in more damage to the surface of the Cu sample, where the top layer is visibly starting to burst.

**Figure 3:**
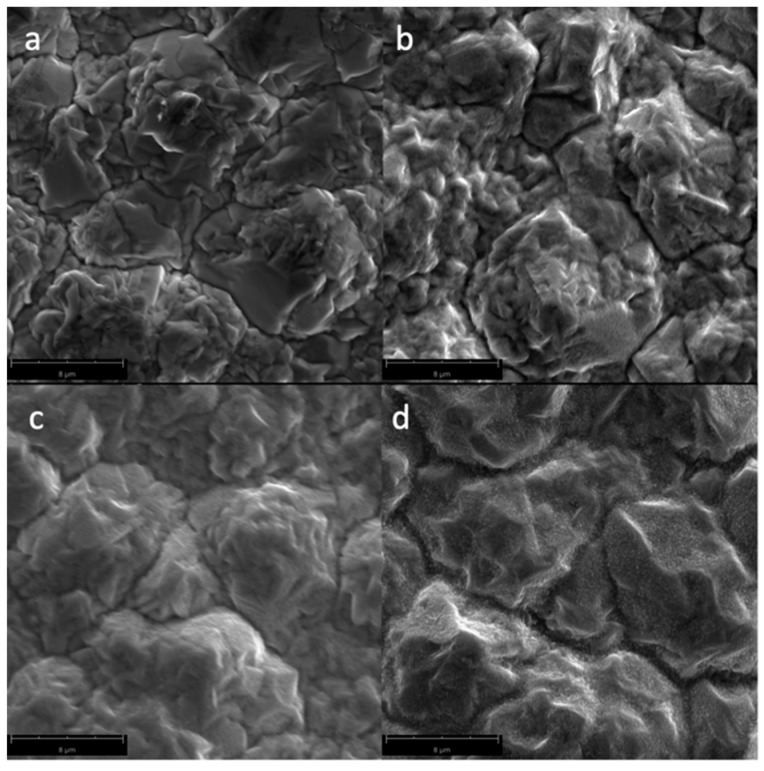
SEM micrographs of the surfaces of the Cu samples (magnified at 10,000×) subjected to oxygen plasma with varying irradiation times: (**a**) untreated; (**b**) 60 s; (**c**) 180 s; and (**d**) 300 s.

Figure 3 shows the SEM micrographs of the Cu samples subjected to oxygen plasma with varying irradiation times. The formation of the CuO nanostructures can be traced as the irradiation time increases. Longer exposure of the Cu sample to the plasma species is necessary to attain the change in surface chemistry which results in the CuO nanostructures. This is due to the high activation energy needed to produce CuO from Cu, or from its CuO_2_ precursor, as shown in Table 2.

**Figure 4:**
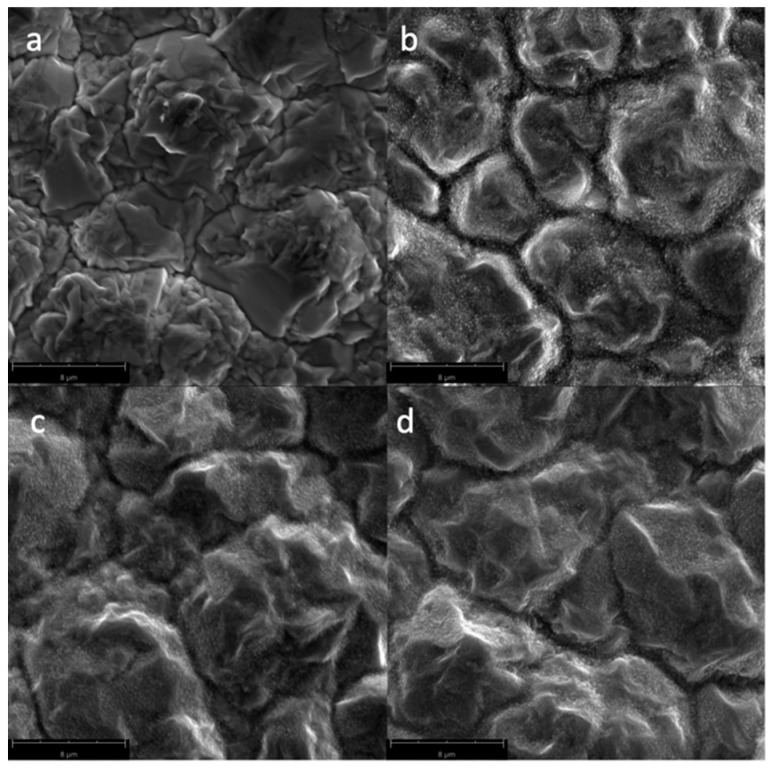
SEM micrographs of the surfaces of the Cu samples (magnified at 10,000×) subjected to oxygen plasma with varying O_2_ gas flow rates: (**a**) untreated; (**b**) 10 sccm; (**c**) 15 sccm; and (**d**) 20 sccm.

Figure 4 shows the SEM micrographs of the Cu samples subjected to oxygen plasma with varying gas flow rates. Since the power and irradiation times were kept constant at 300 W and 300 s, respectively, CuO nanostructures could form, even at the lower gas flow rate of 10 sccm.

**Figure 5:**
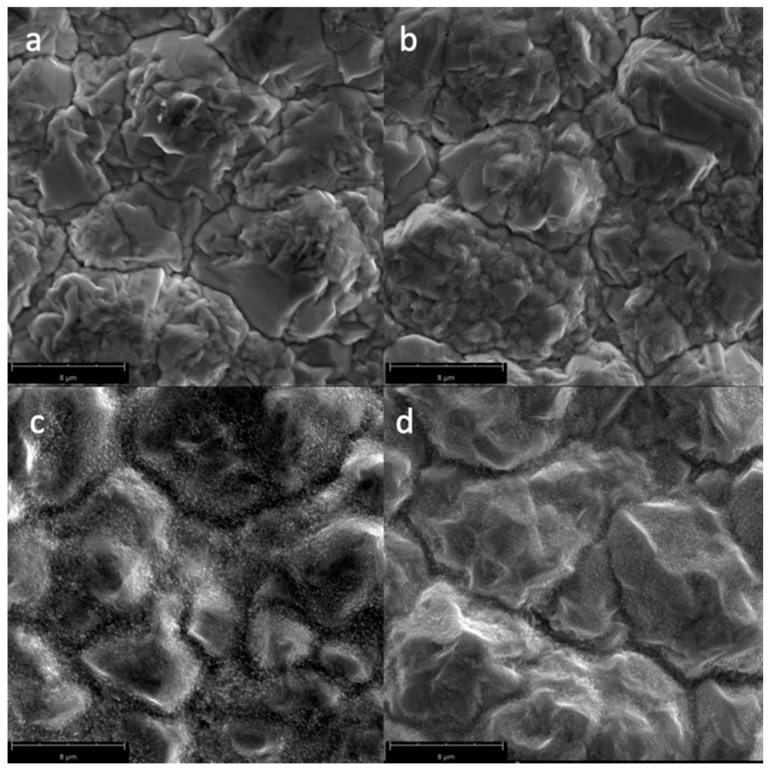
SEM micrographs of the surfaces of the Cu samples (magnified at 10,000×) subjected to varying pulsed oxygen plasmas with duty cycles: (**a**) untreated; (**b**) no pulsing; (**c**) 50%; and (**d**) 80%.

Figure 5 shows the SEM micrographs of the Cu samples subjected to pulsed oxygen plasma with varying duty cycles. The CuO nanostructures are more prominent at the 50% and 80% duty cycle than the 0% pulsing. These observations are in accordance with the water contact angle measurements presented in Section 3.1.

### 3.3. Energy Dispersive Spectroscopy (EDS)

**Figure 6:**
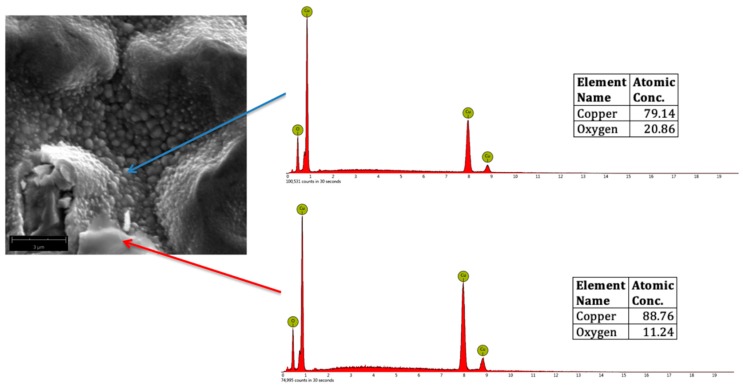
Representative SEM micrograph (magnified at 20,000×) of oxygen plasma-treated Cu, showing CuO (blue arrow) and Cu_2_O (red arrow) with the corresponding Energy Dispersive Spectroscopy (EDS) spectra.

CuO, Cu_2_O, and Cu_2_O_3_ were formed in the surface of plasma-treated samples after analyzing the atomic concentrations of Cu and O from the EDS spectra. Figure 6 shows a representative SEM micrograph of oxygen plasma-treated Cu, showing CuO nanostructures (indicated by the blue arrow) and Cu_2_O (indicated by the red arrow). For reference, the theoretical atomic concentration of CuO is 79.89% Cu and 20.11% O, whereas for Cu_2_O, it is 88.82% Cu and 11.18% O. 

**Figure 7:**
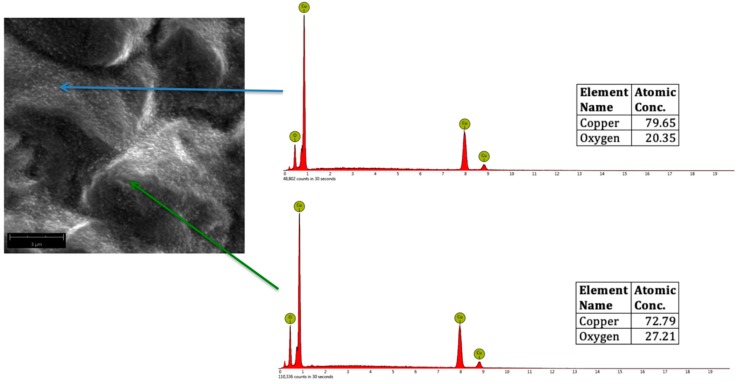
Representative SEM micrograph (magnified at 20,000×) of oxygen plasma-treated Cu, showing CuO (blue arrow) and Cu_2_O_3_ (green arrow) with the corresponding EDS spectra.

Figure 7 shows a representative SEM micrograph of oxygen plasma-treated Cu, showing CuO nanostructures (indicated by the blue arrow) and Cu_2_O_3_ (indicated by the red arrow). For reference, Cu_2_O_3_ is 72.59% Cu and 27.41% O.

**Figure 8:**
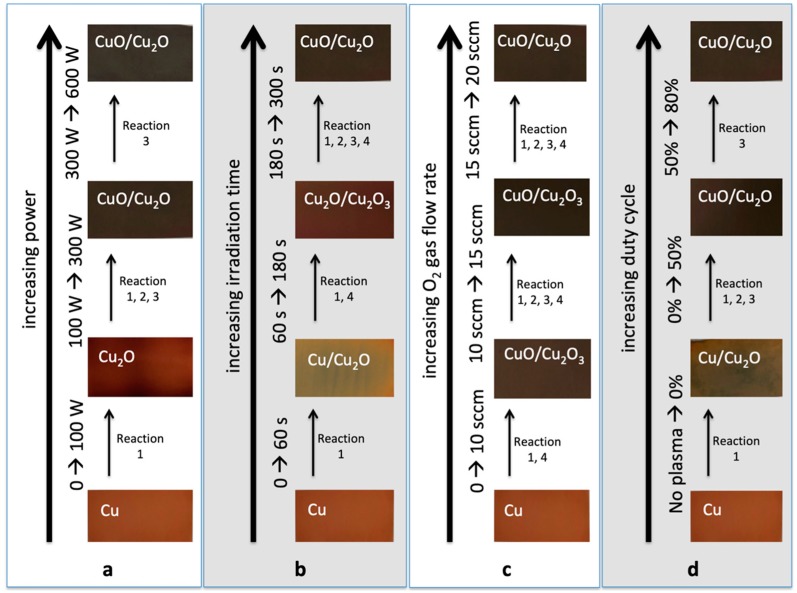
Mechanism of CuO, Cu_2_O, and Cu_2_O_3_ formations from the plasma surface modification of Cu, with increasing: (**a**) power; (**b**) irradiation time; (**c**) O_2_ gas flow rate; and (**d**) duty cycle. The images of the actual samples can also be seen with the corresponding chemical elements and compounds.

Figure 8 summarizes the oxidations of Cu, namely, CuO, Cu_2_O, and Cu_2_O_3_ from the plasma surface modification of Cu, with increasing: (a) power; (b) irradiation time; (c) O_2_ gas flow rate; and (d) duty cycle. The images of the actual samples can also be seen with the corresponding chemical elements and compounds, as confirmed from the EDS spectra. Those with two oxides written on the sample means that both of them existed on the surface of the Cu sample. For example, at the 600 W plasma treatments, both CuO/Cu_2_O is written, which means that CuO nanostructures were found on the surface of the Cu_2_O.

The color of the actual plasma-treated Cu samples also provided evidence on whether CuO nanostructures (usually black in color) or Cu_2_O (usually red in color) had formed on the surface of the material.

Figure 8 also outlines the possible mechanisms on how CuO, Cu_2_O, and Cu_2_O_3_ formed, based on the varying plasma parameters. The model reaction set outlined in Table 2 is used to explain the possible major reactions involved in the O_2_ plasma treatment of Cu.

In Figure 8a, reaction 1 is the most probable reaction that might occur when the Cu is exposed to 100 W plasma. This is seen from the formation of the Cu_2_O which has a lower activation energy compared to the other form of oxides, as explained in Section 3.1. Higher plasma interactions with the sample at higher power enabled reactions 2 and 3 to occur, in order to obtain CuO nanostructures from the Cu_2_O precursor. 

Figure 8b shows the possible reactions that might occur when the irradiation time is increased. At 60 s, the sample showed the formation of Cu_2_O, which has lower activation energy, from Cu. It also shows that before obtaining CuO nanostructures, an intermediate-phase Cu_2_O_3_ was formed, which has an activation energy that is slightly lower than when obtaining CuO from reactions 2 and 3. 

Figure 8c shows the possible reactions that might occur when the O_2_ gas flow rate is increased. Since the power and irradiation time were maintained at 300 W and 300 s, respectively, it shows us that CuO can be formed even at a low gas flow rate of 10 sccm, since the activation energy to create CuO from reaction 1 might have been achieved due to high power and irradiation time. The formation of Cu_2_O_3_ from 10 sccm and 15 sccm was due to the relatively lower pressure compared to 20 sccm.

Figure 8d shows the possible reactions that might occur when the duty cycle of the pulsed plasma is increased. As expected, there were still no CuO formations on the 0% duty cycle, which is in accordance with the discussion in Section 3.1 and Section 3.2.

Increasing the power, irradiation time, gas flow rate, and duty cycle increases the plasma temperature and pressure, which also increases the plasma energy that gets in contact with the Cu sample. These results are in accordance with the study of Choudhary et al. [18] in the oxidation mechanism of Cu from the assisted thermal evaporation technique, where the formation of Cu_2_O was achieved between the temperatures of 150 °C and 320 °C, while CuO can be converted from Cu_2_O at temperatures above 330 °C and longer durations. 

For all the plasma-treated Cu samples, EDS and SEM results show that the effects of the oxygen plasma can be observed from depths of 2.5 μm to 3.0 μm from the surface of the Cu samples. At these thicknesses, complete or partial oxidation of Cu occurred, as evidenced by the formation of the different copper oxides shown in Figure 8. The thicknesses of the copper oxides formed in the surface of the Cu samples do not affect the overall wetting properties observed in the material. This also means that the modification of the Cu sample to attain hydrophilic and superhydrophilic properties is not only due to the nanostructures on the surface, but also the changes in the chemistry of the material. Comparing this study with the research conducted by Li and Hess [33] where Cu films were oxidized using oxygen plasma discharge, the plasma effects on the Cu samples—primarily the thermal oxidation—also increased as the plasma power increased. 

## 4. Conclusions

CuO nanostructures formed from the plasma surface modification of Cu exhibited hydrophilic and superhydrophilic properties from the measured water contact angles of less than 90° or <10°, where the liquid drop was shown to spread on the samples’ surfaces within 1 s of contact. These enhanced wetting properties are comparable with the hydrophilic and superhydrophilic properties observed from the nanostructures present on the leaves of plants such as *Alocasia odora*, *Calathea zebrina*, and *Ruelia devosiana.* The decrease in the water contact angles with increasing power, irradiation time, gas flow rate, and duty cycle can be attributed to the increased surface roughness and the formation of CuO nanostructures, as well as the transformation of Cu to either CuO_2_ or Cu_2_O_3_, as confirmed by the EDS spectra and SEM images. The presence of the CuO nanostructures on the surface of Cu is very useful in terms of its possible applications, such as: (1) in antimicrobial and anti-fouling tubing; (2) in the improvement of heat dissipation devices, such as microfluidic cooling systems and heat pipes; and (3) as an additional protection to the Cu from further corrosion. Increasing the plasma power enhances the formation of CuO nanostructures, since higher plasma power can produce more energetic plasma species, which can easily attain the activation energy needed to produce CuO. The variation in the irradiation time and gas flow rate showed the process from forming CuO from Cu, Cu_2_O, and the intermediate-phase Cu_2_O_3_. The higher activation energy needed to produce CuO nanostructures can be attained at higher duty cycles, due to the increased dissociation in the plasma.

## Figures and Tables

**Table 1:** Summary of the plasma surface modification parameters and the corresponding water contact angle measurements.

Sample		Power (W)	Irradiation Time (s)	O_2_ Gas Flow Rate (sccm)	Duty Cycle (%)	Water Contact Angle (°) (0 Days after Plasma Treatment)	Water Contact Angle (°) (30 Days after Plasma Treatment)	Water Contact Angle (°) (60 Days after Plasma Treatment)
Cu-0 ^a^		Untreated					116 ± 2	
Varying Power	Cu-1	100	300	20	80	22 ± 2	23 ± 3	24± 3
	Cu-2 ^a^	200	300	20	80	<10	<10	<10
	Cu-3	300	300	20	80	<10	<10	<10
Varying Irradiation Time	Cu-4	300	60	20	80	<10	<10	<10
	Cu-5	300	180	20	80	<10	<10	<10
Varying O_2_ Gas Flow Rate	Cu-6	300	300	10	80	15 ± 1	16 ± 2	16 ± 1
	Cu-7	300	300	15	80	<10	<10	<10
Varying Duty Cycle	Cu-8	300	300	20	0	34 ± 1	35 ± 3	36 ± 2
	Cu-9	300	300	20	50	24 ± 1	24 ± 1	25 ± 2

^a^ Cu-O and Cu-2 were also used to compare the effects of the plasma treatment of Cu with varying irradiation times, gas flow rates, and duty cycles.

**Table 2:** Model reaction set with the corresponding activation energy of the products.

Reaction No.	Reaction	Activation Energy (kJ/mol)	Reference
1	4Cu + O_2_ ↔ 2Cu_2_O	111 (T = 600–800°C)	[20]
2	2Cu + O_2_ ↔ 2CuO	191	[21]
3	2Cu_2_O + O_2_ ↔ 4CuO	168 (T < 700°C)	[19]
4	Cu_2_O + O_2_ ↔ Cu_2_O_3_	143 (T = 700°C)	Present work
